# From bedside to bench—practical considerations to avoid pre-analytical pitfalls and assess sample quality for high-resolution metabolomics and lipidomics analyses of body fluids

**DOI:** 10.1007/s00216-021-03450-0

**Published:** 2021-06-22

**Authors:** Rainer Lehmann

**Affiliations:** 1 Institute for Clinical Chemistry and Pathobiochemistry, University Hospital Tuebingen, Hoppe-Seyler-Strasse 3, 72076 Tuebingen, Germany; 2 Core Facility Clinical Chemistry Laboratory, German Center for Diabetes Research (DZD), 72076 Tuebingen, Germany; 3 Department for Molecular Diabetology, Institute for Diabetes Research and Metabolic Diseases of the Helmholtz Zentrum Muenchen at the University of Tuebingen, University of Tuebingen, 72076 Tuebingen, Germany

**Keywords:** Metabolomics, Lipidomics, Pre-analytic, Blood, Urine, Cerebrospinal fluid, Serum, Plasma

## Abstract

The stability of lipids and other metabolites in human body fluids ranges from very stable over several days to very unstable within minutes after sample collection. Since the high-resolution analytics of metabolomics and lipidomics approaches comprise all these compounds, the handling of body fluid samples, and thus the pre-analytical phase, is of utmost importance to obtain valid profiling data. This phase consists of two parts, sample collection in the hospital (“bedside”) and sample processing in the laboratory (“bench”). For sample quality, the apparently simple steps in the hospital are much more critical than the “bench” side handling, where (bio)analytical chemists focus on highly standardized processing for high-resolution analysis under well-controlled conditions. This review discusses the most critical pre-analytical steps for sample quality from patient preparation; collection of body fluids (blood, urine, cerebrospinal fluid) to sample handling, transport, and storage in freezers; and subsequent thawing using current literature, as well as own investigations and practical experiences in the hospital. Furthermore, it provides guidance for (bio)analytical chemists to detect and prevent potential pre-analytical pitfalls at the “bedside,” and how to assess the quality of already collected body fluid samples. A knowledge base is provided allowing one to decide whether or not the sample quality is acceptable for its intended use in distinct profiling approaches and to select the most suitable samples for high-resolution metabolomics and lipidomics investigations.

**Graphical abstract:**

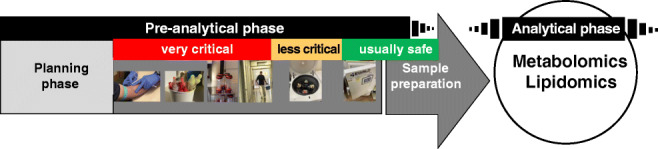

## Introduction

In targeted and non-targeted biomedical metabolomics and lipidomics studies, the quality of the achieved analytical data is not only dependent on the knowhow and experience of (bio)analytical chemists, but is highly dependent on the quality of the sample. Low-quality samples caused by inaccuracies in the pre-analytical phase are the reason for up to 80% of laboratory testing errors in daily clinical routine diagnostics [1–3]. Covering a multitude of lipids and other metabolites of extremely different stabilities, the sample quality is very critical for high-resolution approaches, which is in contrast to targeted analysis of selected distinct parameters in clinical chemical diagnostic routine. So, accidental or systematic pre-analytical issues affecting compounds with low stability can lead to high variability in the analytical data or even bias final results and conclusions.

**Fig. 1:**
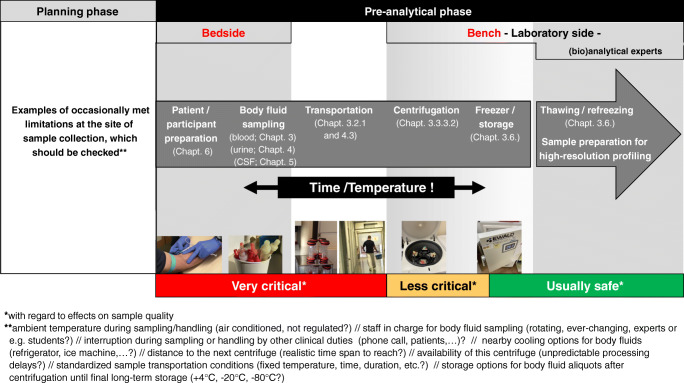
Flowchart of the pre-analytical phase from bedside to bench in biomedical metabolomics and lipidomics projects. Risk assessment was applied solely with regard to negative effects on sample quality (multistage metabolite and lipid extraction is not considered)

It is important to note in this context that the most critical steps regarding the quality of body fluid samples take place in the clinical settings or the animal house, but not in the laboratories of analytical (bio)chemists or biobanks where all processes are usually highly standardized and strictly controlled to avoid any deterioration of sample quality (Fig. 1). In many clinical settings, the collection of study samples is a compromise between requirement and feasibility. Hence, (bio)analytical chemists should be aware that the samples, although entitled to be of high quality, may have some limitation with respect to specific needs essential for high-resolution analysis. This stands in contrast to the usual statement that sample collection is the easy part in complex biomedical biomarker studies. Figure 1 shows major steps of the pre-analytical phase including cross references to the corresponding chapters, as well as examples of probable limitations at the site of sample collection.

This review will discuss key aspects of the pre-analytical phase, such as collection of body fluids (blood, urine, cerebrospinal fluid), sample handling, transportation, and storage in freezers. It will provide a base for identification of potential pre-analytical pitfalls already in the planning phase and enable to assess the quality of already collected body fluid samples. This information should facilitate the decision to select the most appropriate sample for each profiling approach, since even the best high-end instruments and/or greatest analytical experience and efforts of (bio)analytical chemists cannot compensate effects of pre-analytical inadequacies and errors. Best practice strategies and potential pitfalls of the pre-analytical multistage body fluid processing for lipid and metabolite extraction are discussed in detail elsewhere [4–6] and will not be considered in the context of this review.

## The planning phase in a biomedical metabolomics and lipidomics study

The planning phase is an important first pre-analytical step, although it takes place before sample collection. At this point, future limitations and errors affecting sample quality should be identified, jointly discussed by all involved scientists, i.e., (bio)analytical chemists, study nurses, clinical doctors, etc., and subsequently eliminated. Table 1 summarizes notes and considerations for those discussions. Usually, a mismatch exists between the idea of ideal sample collection conditions and actual logistic and practical possibilities, respectively limitations. This implies the need for compromises and well-balanced joint decisions which are finally committed in recommendations and standard operating procedures (SOPs) to ensure collection of good-quality samples for metabolomics and lipidomics analyses. It means agreeing on sample collection and handling procedures that are practical and achievable by all parties. The following chapters highlight all the critical pre-analytical steps with respect to most common errors and how to avoid them, and offer as well as suggestions and recommendations for solutions on how to get high-quality body fluids from the bedside to the bench for metabolomics and lipidomics analyses.

**Table 1:** Notes and considerations for the planning phase

Materials - check suitability of all tubes and tips (chemical resistance, contaminations, etc.) - in multi-center studies: - use same brand and type, i.e., material, additive(s), etc. - harmonize sample labeling for all participating sites - if different brands are unavoidable test all sample collectors, tubes, … - tube labels: do they withstand all storage conditions (e.g., −196 °C of liquid nitrogen)?	
Study participants - prepare a comprehensible participant instruction and meaningful study questionnaire - standardize participant conditions - fasting period (≥ 12 h) - resting period (no unaccustomed or strenuous physical activity 48 h before collection) - day time for sample collection (ideally between 7 and 10 am) - health state of control groups: perform simple clinical chemical test and record results - some aspects to be considered in a study questionnaire - anthropometric data - stress before sample collection - unaccustomed situations the day before sample collection (extreme exercise, food excess,…) - xenobiotics, dietary supplements, drugs - life style factors (e.g., alcohol consumption per day or week, cigarettes per day,…) - food or special food the day/evening before specimen collection - special diets (e.g., Atkins diet, vegan, …)	
Storage of the metabolomics and lipidomics data - define precisely data storage architecture (e.g., file and sample name), access privileges, and data back-up	
Body fluid collection and preparation - apply uniform sample labeling - define sample type and collected volume - plasma or serum (which additive)? - midstream second morning urine or 24-h collection urine? - standardize collection procedure (e.g., tourniquet application time, site of venipuncture, collection order for different tubes, sample mixing, …) - sample handling and transportation: standardize time period and temperature until centrifugation (continuous cooling is highly recommended) - processing delays: define acceptance criteria regarding sample stability - define centrifugation conditions for blood, urine, CSF (G force, temperature, time, brake use, etc.) - standardize the post-centrifugation period until storage or further processing (should be as short as possible, but fulfillable) - define volume and number of sample aliquots for long-term storage at −80 °C or below by the analytical needs - thaw samples at 4 °C and standardize accurate mixing of thawed samples (avoid repetitive freeze-thaw cycles and mark refrozen samples) - deviations from the protocol: specify recording and documentation (information should be accessible to all involved scientists)	

## Blood samples

### Collection tubes may introduce chemical noise

Nowadays, venipuncture tubes are generally made of plastic rather than glass. Two main types of tubes are commonly in use in hospitals, either containing a polymer-based gel which separates cellular compounds from plasma or coagulum from serum after a centrifugation step, or tubes without such a separator between serum/plasma and cellular compounds. Additionally, tubes contain usually additives, either an anticoagulant to prevent clotting (plasma tubes) or a clotting promotor to reduce the coagulation time of whole blood (serum tubes).

The release of plasticizers from blood collection tubes into samples is a potential issue that may have a particularly negative impact on high-resolution mass spectrometric analysis. Furthermore, additives in tubes may contain interfering compounds [7], e.g., spurious amount of sarcosine in one brand of EDTA vacutainer tubes [8]. Tubes containing gel-based separators should be used with caution due to the risk of improper gel barrier formation [9, 10] or alteration of the concentration of metabolite groups of interest or distinct compounds by the gel, e.g., methionine sulfoxide [10].

This means that during the planning phase of a project, the blood collection tubes to be used should be tested to determine whether they are suitable or whether they contain or release interfering compounds (Table 1). A detailed test description tubes can be found for example in the method section of reference [7]. The suitability check also applies to all other plastic tubes and compounds planned to be used in the pre-analytical process up to sample preparation for mass spectrometric analysis, i.e., for all types of liquids (e.g., solvents), pipette tips and other plastic products, with regard to short- and long-term sample storage in freezers or liquid nitrogen, the suitability of cryotubes or other vials and the corresponding labels (Table 1). Special attention is needed when long-term freezing at ultra-low temperatures is planned, because not all labels and markings endure temperatures of −80 °C or −196 °C, which in the worst case can lead to detached labels.

### Whole blood

#### From blood drawing until separation from cells is most critical for plasma and serum quality

**Fig. 2:**
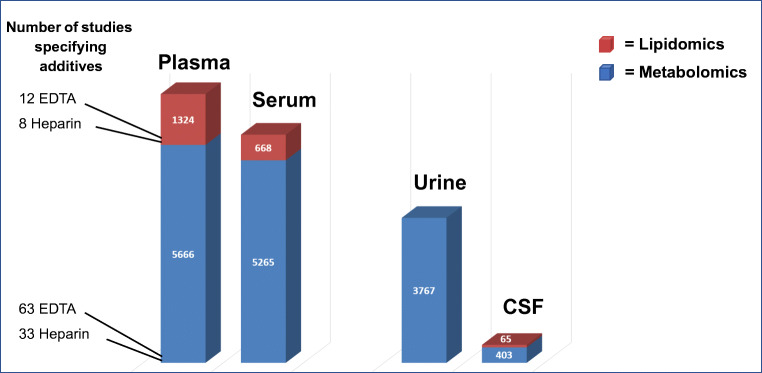
Number of biomedical metabolomics and lipidomics publications studying major body fluids (blood, urine, cerebrospinal fluid (CSF)) based on a PubMed search (dated Feb. 2021). Search terms: metabolomics and plasma; metabolomics and serum; lipidomics and plasma, etc.). The number of specified additives in plasma samples is also given (search terms: metabolomics and plasma and EDTA, etc.)

Blood is the most commonly analyzed body fluid in biomedical metabolomics and lipidomics studies (Fig. 2). In a sequence drawing blood in different tubes for various analytical purposes, the question arises which is the best sampling order, particularly which position is best for the tube for metabolomics and lipidomics. Up to now, no data exist to answer this question, but the tube for metabolomics and lipidomics should not be the first one, at least if the collection of plasma is intended (for details, see the “Usable and unusable anticoagulation additives” section and Table 2). After blood sampling, nearly half of the volume in the tube consists of cellular compounds, and the other part is serum or plasma, depending on the collection tube that is used. These billions of cells are highly active in the anaerobic milieu of the tube, continuously releasing, up taking, and metabolizing metabolites. Detectable effects on the metabolome depend mainly on the interval until erythrocytes, leucocytes, and platelets are separated from plasma or serum and on the temperature during this time period [11–13]. For example, lower temperatures reduce the activity of cellular metabolism. Hence, important questions that (bio)analytical chemist should ask the clinical cooperation partner are the time frame from blood drawing to centrifugation and the temperature during this period.

**Table 2:** Suggestions of specific procedures to minimize pre-analytical issues in metabolomics and lipidomics studies respecting compromises between requirements for perfect sample quality and practical feasibilities at the medical sites

	Blood	Urine	Cerebrospinal fluid (CSF)
Pre-tests	Testing of the suitability of collection tubes, tips, cryovials, etc. (chemical noise, etc.)		
Sample labels on tubes	Definition of reasonable and comprehensible sample labeling		
File names for data base storage	Definition of reasonable, comprehensible, and uniform sample and file names		
Study control group	Exclude apparently healthy subjects		
Sample material	EDTA plasma* (should not be the first tube in a blood drawing sequence) Cooled at once (4–8 °C) after collection	Midstream 2^nd^ morning urine (standardize or, at least, record the diet on previous day)	Pairs of CSF and EDTA plasma**
Transportation	At 4–8 °C (cold pack or iced water)		
Centrifugation and aliquoting	Within 2 h after collection		
	2500×*g*/4 °C/10 min/using brake	2000×*g*/4 °C/10 min/using brake	CSF: 2000×*g*/4 °C/10 min/using brake
After centrifugation	Transfer supernatant aliquots into cryotubes as soon as possible (keep samples always at 4 °C until freezing)		
Storage	−80 °C or below		
Thawing	At 4 °C (avoid refreezing)		
Recording	Documentation of any deviation from the protocol		

*First, fill a 5-ml discard tube to avoid possible thromboplastin contaminations if no other blood drawing is intended

**Collected at the same point of time

Drastic minimization of the time until centrifugation is often difficult to achieve in clinical setting and animal houses due to logistic limitations. Access to a centrifuge is often not possible at the site where the blood is drawn. A possible strategy to manage this problem is to stabilize the composition of the metabolome and lipidome in whole blood by reducing the temperature in the blood collection tube after drawing and during transportation [11, 12, 14–16]. Based on our experience, an achievable time frame until centrifugation is 120 min at 4 °C, which can be fulfilled in most clinical settings and is also acceptable with respect to sample quality (0.5% significant changes of metabolite features) [11] (see also Table 2).

Immediate cooling after blood drawing (by cold packs, in refrigerator, or in iced water) is highly advisable to avoid great fluctuations in the results or even misleading findings [7, 14, 17–19]. Unfortunately, this is only possible for plasma blood collection tubes, because the clotting process for generating serum from whole blood requires a defined time at room temperature (for details, see the “Choosing plasma or serum? Metabolite and lipid profiles are different” section). Accordingly, it is much easier to standardize the generation of plasma and reduce any release of enzymes or ex vivo cellular metabolic activities after blood drawing. For EDTA plasma, we detected in a non-targeted metabolomics approach that only 0.5% of more than 1800 ion masses showed significant alterations within 120 min when whole blood is cooled down to 4 °C immediately after drawing [11]. In contrast, almost 10% of all metabolite features changed significantly at room temperature [11]. At some sample collection sites in hospitals, it is not possible to fulfill even a time frame of 2 h until centrifugation. Therefore, we also checked a longer time frame of 480 min exposure of whole blood to 4 °C. We found less than 2% significant changes in the metabolome, compared to more than 16% altered ion masses at room temperature during the same period [11]. After 48 h at room temperature, more than 30% of 1012 tested metabolites in EDTA whole blood had changed. The most affected were nucleotides, energy-related metabolites, peptides, and carbohydrates [12]. In an elegant study, the group of M. Giera studied the pre-analytical stability of 133 oxylipins reporting no alterations beyond the analytical variance of 20% in EDTA whole blood for up to 24 h at 4 °C (or at 20 °C for 4 h) [14]. In addition to refrigeration, the authors suggested after centrifugation of whole blood to add either methanol [20] or butylated hydroxytoluene [14] into EDTA plasma for lipidomics investigation, or at least for the profiling of oxylipins. Cooling of whole blood activates platelets. However, in the metabolite profile covered by our recent approach, we could not detect a relevant effect of platelets after cooling [15]. If we are given a choice in the planning phase of a biomedical study, we suggest collecting plasma for mass spectrometry-driven metabolomics and lipidomics investigations (kept cooled immediately after blood drawing until centrifugation) [15].

Overly demanding blood processing recommendations, i.e., centrifugation of samples as soon as possible or within a time that is hardly achievable, should be avoided because they may cause variabilities in blood processing until centrifugation. The frequency of such pre-analytical pitfalls, such as difference in time until centrifugation between 15 and 90 min, or even more, depends on the respective additional duties of study nurses or medical doctors. Therefore, it is mandatory to standardize the treatment of whole blood in a feasible manner and to document every accidental or systematic error in order to detect and eliminate possible outliers at an early stage. Table 2 summarizes suggestions of specific procedures to minimize pre-analytical issues in metabolomics and lipidomics studies. The suggestions in this table are based on compromises between requirements for perfect sample quality and practical feasibilities at the medical sites. The best way to minimize the risk of inaccuracies and errors is to organize sample collection in the clinical surrounding at a study ward by a specialized and diligent team focused on specimen collection, cold chain, and blood processing [11].

Exact planning and valid information about the conditions of whole blood handling is imperative for the success of metabolomics and lipidomics studies. This avoids the identification of “biomarkers” reflecting the ex vivo metabolism of red and white blood cells or thrombocytes after blood drawing instead of real biomarkers reflecting the pathophysiological situation of the patients of the study.

#### Effects of hemolysis on metabolite and lipid profiles

**Fig. 3:**
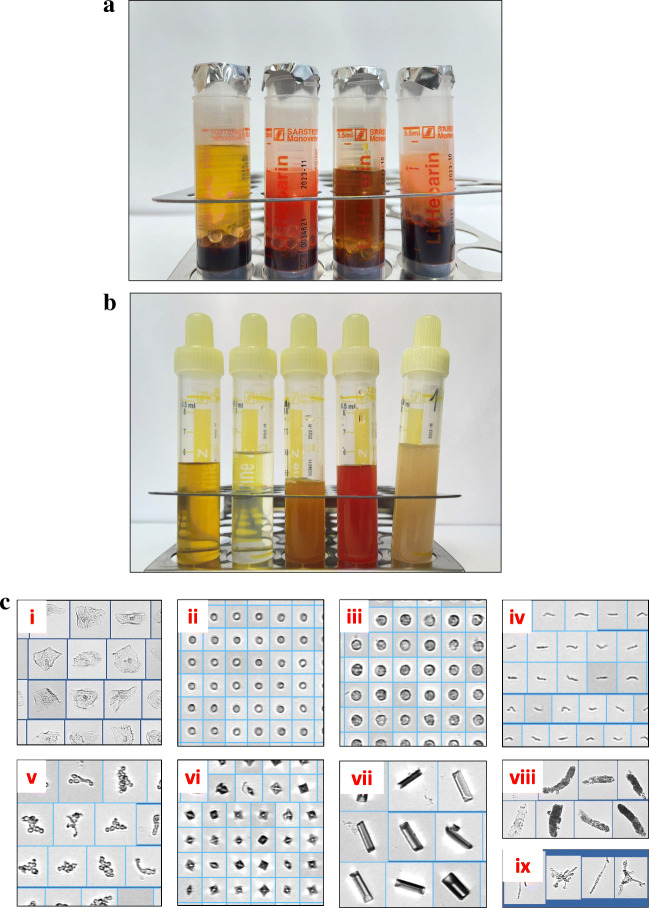
Examples of daily blood and urine samples obtained for routine diagnostics in our hospital. **A** Plasma samples from different patients. From left to right: normal, hemolytic, icteric, and lipemic plasma (only hemolysis negatively impacts the composition of metabolomes and lipidomes). **B** Urines of different patient before centrifugation (two normal urines are shown on the left-hand side). Note: after centrifugation, all urines appear clear and look very similar. **C** Examples of metabolic active cells and other compounds in urines of different patients. (i) Squamous epithelial cells (appearing in normal spot urine, if no midstream urine was sampled), (ii) erythrocytes, (iii) leukocytes, (iv) bacteria, (v) yeasts, (vi) oxalate crystals, (vii) triple phosphate crystals, (viii) granular casts, and (ix) pseudohyphae

In the clinical routine laboratory, sample analysis may be interfered with or biased by extreme concentrations of various compounds. Most common factors, such as hemolysis, icterus, and lipemia, are illustrated in Fig. 3A. However, among these factors, only hemolytic samples cause frequent problems in metabolomics and lipidomics analysis [7, 17, 21]. Dependent on the extent of hemolysis, it could lead to a massive release of proteins (incl. enzymes), as well as metabolites, electrolytes, and lipids into serum or plasma. It may occur in vivo, caused by a disease, or ex vivo caused during blood drawing or whole blood sample handling. In a daily clinical practice, but also in research studies, ex vivo hemolysis is one of the most frequent pre-analytical errors [22]. A variety of reasons may cause ex vivo hemolysis of various stages [23], like lack of experience in blood sampling from a young study nurses or medical students, cooling the sample below 4 °C during transportation, rigorous shaking instead of slightly swaying the tube for mixing additives and whole blood, too low inner diameter of the needle or too strong aspiration of blood, as well as limited time or stress during blood drawing [24].

Notably, hemolytic plasma or serum is not necessarily detected visually, since slightly hemolytic samples look much alike normal samples, but the metabolome has already changed [7, 17, 21]. Hemolysis may affect up to 20% of all features in non-targeted metabolomics approaches [7].

How to reliably identify hemolytic samples? A specific metabolite biomarker or pattern to reveal hemolytic serum or plasma samples directly in metabolite or lipid profiles, i.e., without additional analysis, would be desirable. However, there are no reports published to date, regarding the simplification to detect one of the most common pre-analytical errors. An existing possibility is a routine optical method performed at two wavelengths that allows reliable quantification of free hemoglobin in plasma or serum [23].

### Plasma and serum, a pre-analytical point of view

#### Choosing plasma or serum? Metabolite and lipid profiles are different

Plasma and serum are almost equal in use for metabolomics investigations of blood (Fig. 2). These data are in line with the still ongoing debate of (bio)analytical chemists which sample material is more suited for profiling approaches. For historical reasons, serum is the first-choice sample material in many clinical studies and consequently stored in huge numbers in biobanks, although the majority of high-throughput routine diagnostic assays meanwhile prioritize plasma. This means that the use of serum for metabolomics investigation is sometimes based on the fact that serum is the only available sample material. But what should be considered when a (bio)analytical chemist has the choice to select either plasma or serum for high-resolution analysis?

The principal difference between serum and plasma is the initiation of a clotting process (serum) or its prevention (plasma) inside the blood collection tube after drawing the blood. This difference involves consequences for the corresponding metabolite profiles. During the clotting process to generate serum out of whole blood, which imperatively need the exposure to room temperature, platelets and enzyme cascades are activated and platelets release proteases and other enzymes as well as metabolites, including lipids [15, 25]. For the generation of plasma, this clotting process is prevented by anticoagulation additives inside the tube. Based on the described essential difference in the generation of plasma and serum, it can be easily considered that the metabolome and lipidome between serum and plasma is different. In general, metabolomes and lipidomes in serum show a higher variability [26], and the levels of most affected metabolites and lipids are higher in serum [8]. In paired serum and EDTA plasma samples, a metabolite profile composed of 216 metabolites showed for almost half of all metabolite levels significant differences between these two sample materials in a mass spectrometric-driven targeted metabolomics approach [15]. Another study found even more obvious significant differences (104 out of 121 metabolites) when serum and plasma were compared [27]. Nine of these metabolites showed 20–50% higher concentrations in serum [27]. The group of M. Holcapek reported recently in a lipidomics approach 20% higher lipid levels in serum in comparison to plasma [28]. As a consequence, it is not easy to deduce findings from serum to plasma and vice versa without verification in the corresponding sample material.

#### Serum

##### Check tubes for chemical noise

Clotting of blood after drawing into a tube occurs spontaneous by surface activation of the hemostasis systems. After the end of the clotting process, serum can be collected subsequent to centrifugation and separation of the coagulum. Hence, since many decades, serum was the sample material of choice to study the cell-free part of blood. Even today, commercial serum blood collection tubes containing no additives are still available, but the clotting time until centrifugation of around 60 min exposure at room temperature is quite long. Nowadays, nurses and medical doctors prefer tubes containing coagulation enhancer as additives to standardize and shorten the clotting process. These enhancers could either be silicate-based resulting in clot formation after 20–30 min exposure to room temperature, or clotting is enhanced by the addition of the enzyme thrombin allowing a clotting time of 5 min, but these tubes are 10-times more expensive than silicate containing blood collection tubes. Depending on the way the coagulation process was initiated, i.e., by silicate or by thrombin, significant differences in the metabolome between these kinds of serum samples are detectable [15].

Different brands of serum collectors are available. Therefore, it is recommended to test the suitability of the intended serum collection tubes used for high-resolution metabolomics and lipidomics analysis (Table 1). We observed that serum collection tubes used in our university clinic, which contain kaolin-coated plastic beads, release polymers leading to interfering clusters in metabolomics mass spectra of serum [7]. In such case, the only way out is to test and use tubes from other companies (not very appreciated by the people in the hospital drawing the blood) or to choose serum tubes from the same brand with different formulation or without additives (leading to a non-standardized clotting process).

##### Cooling at once after blood drawing is not possible

A specific characteristic of the collection of serum samples is the need for an accurate and standardized coagulation at room temperature before sample cooling. Variabilities in ambient temperature and/or clotting time during this process may alter the corresponding metabolomes. Since in clinical settings not much attention is payed to the clotting process of a sample after blood drawing, it is useful to check the conditions and common duration in the planning phase of a study (see also Tables 1 and 2). Alterations in the levels of lipids and other metabolites are in particular caused by the activity of platelets in each individual sample. Platelets are directly involved in the coagulation process during the generation of serum at room temperature. Standardization of the clotting process (brand of tubes, kind of coagulation enhancer, clotting time, and ambient temperature) is mandatory (Tables 1 and 2). Samples from multi-center studies represent a major challenge in this context [28]. The possibility using different clotting additives in the tube, differing clotting protocols, etc., is quite possible at the different sites of a multi-center study. NMR and mass spectra show alterations of the serum metabolome when the clotting time differs between sample sets [15, 29]. After separation of the clot from serum by centrifugation, the metabolome in serum samples, as well as plasma, is almost stable, i.e., showing even at room temperature only minor change after several hours [6, 30, 31].

#### Plasma

##### Usable and unusable anticoagulation additives

The difference between plasma and serum samples is the inhibition of blood clotting by anticoagulating additives inside the plasma blood collection tube. Therefore, if no other blood drawing is intended in a blood drawing sequence, fill a 5-ml discard tube first to avoid possible thromboplastin contaminations (Table 2). Most common anticoagulants, at least in daily medical routine, are heparin, ethylenediaminetetraacetic acid (EDTA), citrate, and EDTA fluoride. They are complexed with cations like sodium, potassium, ammonium, or lithium. In metabolomics and lipidomics studies, applying mass spectrometric investigations either heparin [6, 28, 32] or more often EDTA plasma [7, 11, 17] is recommended. For NMR-based investigations, EDTA plasma causes interferences in the spectra and is therefore not suitable [33].

Using EDTA- or citrate-containing tubes in reversed-phase-driven LC-MS approaches, (bio)analytical chemists should be aware that at very early elution times, suppression and enhancement effects on the ionization of polar metabolite co-eluting with anticoagulation additives, like EDTA or citrate, may occur, demonstrated in an elegant study by Barri and Dragsted [32]. The broad elution peak of citrate or EDTA affected the ionization efficiency of distinct compounds, like uridine, methionine, adenine, L-pyroglutamic acid, and arginine [32]. On the other hand, the ionization of creatinine, proline, and valine showing retention in the same time window was unaffected. Furthermore, based on the cations complexed with the additives, the increased occurrence of respective adducts should be respected [32]. Recently, high similarity in lipid levels and profiles between EDTA and lithium-heparinate plasma was demonstrated [28]. In contrast, Hahnefeld et al. reported in a comparison of lipid profiles collected in either EDTA or sodium fluoride/citrate tubes a markedly increased signal level of some endocannabinoids and arachidonoyl ethanolamide in EDTA tubes [19]. For targeted approaches, Paglia and colleagues reported the detection of spurious amount of sarcosine in a brand of EDTA vacutainer tubes and advise against the use of these tubes for the exact quantification of sarcosine [8].

Lithium heparin is another common anticoagulation additive in commercial blood collection tubes. Several groups prefer and recommend the use of Li^+^-heparin tubes [6, 28, 32]. However, an example of own experience of brand-specific effects of tubes was the detection of prominent polymer clusters in mass spectra from Li^+^-heparin tubes during a suitability test of all blood collection tubes routinely used in our university hospital (all originate from the same manufacturer) [7]. Subsequent testing of Li^+^-heparin tubes from another leading company did not show any disturbance in the total ion chromatogram (unpublished observation). The tested brands were the two world leading manufacturers of blood collection tubes. This underline, as noted in Table 1, the absolute need to test every specific type of sample collection tube before the use in biomedical projects to exclude in targeted approaches any non-tolerable interference on compounds of interest or in non-targeted approach more general effects on the analytical metabolite profiles (Table 1).

It is quite important to fix all specific information about tube type (including order number) in the pre-analytical SOP and communicate it clearly to the persons collecting the blood samples. After the plasma samples had arrived in the freezer, it is too late. Special care is required in multi-center studies because, even though it is possible to arrange collection of the same type of plasma, e.g., K+-EDTA plasma, at each participating hospital, it is highly unlikely that all sites will use the same brand of sample collector.

In this context, two aspects have to be considered: (a) additives are inevitable, and (b) analytical pre-tests focusing on the compounds of interest are needed before start of a biomedical metabolomics/lipidomics project to figure out the most suitable blood samplers. An intense literature search could also be an option, but most publications lack a clear specification of the used blood sample collector and brand (Fig. 2), even the naming of the included additive in the investigated plasma samples is not common practice. In a PubMed search (dated Feb. 2021), the huge majority of the more than 5600 biomedical metabolomics publications that analyzed plasma did not further specify the sample material (only 63 specified the use of EDTA and 33 Li^+^-heparin plasma). The same was found in lipidomics studies: of more than 1300 reports, only 12 specified the use of EDTA and 8 Li^+^-heparin plasma (Fig. 2).

##### The centrifugation force of whole blood can alter the metabolite profile in plasma

A less required information in the planning phase or during the selection of plasma samples from a biobank is the applied centrifugation force on whole blood for the separation of erythrocytes, leukocytes, and platelets. Excessive forces (> 4500*g*) carry the risk to cause hemolysis and they are usually avoided. However, if too low a centrifugation force or too short centrifugation time is applied, improper separation of cells from plasma may occur.

Common centrifugation forces for whole blood used in clinical routine laboratories and biobanks worldwide are between 1500 and 4000*g* for 5–10 min. Erythrocytes and leukocytes can be removed under these conditions, but a significant number of platelets still remain in the EDTA plasma fraction. For instance, at conditions of 1,500*g* for 10 min, 137,500 platelets per microliter remain in EDTA plasma [34]; at 3,000*g* for 5 min, 59,500 platelets per microliter [34]; and at 4000*g* for 10 min, 27,000 remaining platelets per microliter were detected [15]. Hence, even state-of-the-art separated plasma is not cell-free. The correct term for this standard plasma is “platelet-poor” plasma (PPP). Generation of platelet-free plasma (PFP), which contains per definition < 10,000 platelets per microliter, needs more complex centrifugation steps that are usually not applied in clinical routine laboratories or biobanks.

Regardless of whether a platelet-poor standard plasma sample is frozen directly for storage or processed for immediate metabolomics analysis, the platelets contained in the sample are lysed, thereby releasing membrane lipids and cellular content into plasma. Fortunately, in a comparison of different EDTA plasma samples obtained by a centrifugation of 1,500*g* for 10 min (137,500 platelets/μl) vs 3,000*g* for 5 min (59,500 platelets/μl), the alterations of the metabolome showed only slight differences in a PLS-DA analysis of NMR and LC-MS spectra [34]. A comparison of platelet-free and platelet-poor EDTA plasma (i.e., plasma containing 3,000 vs 27,000 platelets/μl) revealed no significant differences in a metabolite profile of 216 metabolites [15]. Current data suggest that plasma generated with low centrifugation force (e.g., at 2000*g* or below) may bear a risk for possible alterations in the metabolome or lipidome by platelet contaminations, which can be increased by additional short centrifugation time. Another aspect in this context is that in extreme cases in critically ill patients, the individual platelet count varies and range from less than 10,000 to more than 1,000,000 platelets/μl [25].

In summary, parts of the metabolite or lipid profiles in plasma may be slightly affected by compounds originating from platelets remaining in the sample. The number of remaining platelets in plasma is dependent on the applied centrifugation conditions of whole blood (force and time) and, therefore, it is advisable to standardize and control centrifugation (for suggestions see Table 2). Mixing of plasma derived from different or unknown centrifugation conditions should be avoided. Whenever possible, multi-center studies should apply the same or at least similar centrifugation conditions for whole blood to avoid differences in the metabolome that reflect sample handling at respective sites. For metabolomics and lipidomics, investigations preferred higher centrifugation (between 2300 and 4000*g* for 5–10 min). However, at higher centrifugation forces, the risk of damaging cellular compounds increases, which is especially true for samples from patients with hematological disorders. These diseases may affect the integrity of cellular membranes. In such cases, a compromise is needed for the generation of plasma samples for metabolomics and lipidomics analysis by applying lower centrifugation forces. Table 2 summarize suggestions of specific procedures to minimize pre-analytical issues in metabolomics and lipidomics studies, thereby taking compromises into account between requirements for perfect sample quality and practical feasibilities at the medical sites.

### Dried blood and dried urine spot samples need special considerations

For decades, newborn screening has been performed using dried blood spot (DBS) samples. Among the screened genetic disorders are also metabolic diseases; therefore, DBS-based metabolite profiling of newborn samples is very common for routine diagnostic purposes [35]. Hence, the transition of DBS samples from well-established routine metabolite profiling to the rather new scientific field of metabolomics was small [36]. In addition to DBS, metabolomics analyses of dried urine spot (DUS) samples are also performed [36].

Usually, approximately 20–50 μl of blood is spotted onto a filter paper and dried. For metabolomics investigations, the major difference from plasma or serum analysis is that the metabolite profile of a DBS sample represents not only metabolites from plasma or serum but also from all cellular compounds like erythrocytes, platelets, and leukocytes, i.e., it reflects the metabolome of a lysate of whole blood [37]. Limits of pre-analytical stability of metabolites on the dried filter paper detected by routine newborn screening after long-term storage, such as profiles of acylcarnitines and amino acids, have been well investigated and led to the recommendation to correct quantitative DBS data appropriate for sample decay during long-term storage [38, 39]. Using complementary HILIC and reversed-phase LC-MS to collect thousands of features, Palmer and colleagues studied the stability of the metabolomes in DBS and DUS samples in a non-targeted metabolomics approach [37]. At room temperature (21 °C), metabolite profiles on DBS and DUS are stable for up to 4 weeks and at −20 °C for 1 year. The authors recommended the transportation of DBS and DUS samples within 28 days at room temperature (21 °C) for final storage at −20 °C or −80 °C [37]. In general, preliminary DBS and DUS stability tests of metabolite profiles of interest or distinct metabolites are advised [36, 40], as well as filter paper tests to exclude any interfering compounds released from the filter during sample pretreatment.

### Biomarkers to assess the quality of plasma and serum samples

Every blood sample intended for research use ends up in either plasma or serum aliquots. Therefore, it is not surprising that numerous publications exist investigating the alterations of metabolite or lipid profiles and corresponding quality markers in plasma or serum, i.e., already after the separation from cellular components [30, 41–46]. However, the described changes in serum and plasma are of minor pre-analytical importance for the following reasons: (a) in comparison to changes that occur in whole blood during handling in the hospital, i.e., before the separation of plasma or serum, these alterations are small (far below inter-individual variations), and (b) shortcomings in handling of plasma or serum are not very frequent because the processing of these aliquots usually occurs in clinical laboratories or biobanks under highly standardized conditions, performed by experts focused on sample processing. Therefore, in one of the most critical phases for the quality of plasma and serum samples, i.e., the time period from blood drawing until centrifugation, sophisticated SOP and joint interdisciplinary discussions and agreements with the cooperation partners collecting the blood samples are needed, but they do not guarantee the prevention of either random or systematic deviations in the handling of whole blood.

Quantitative measures of one or more pre-analytical biomarker(s) to assess handling and processing of whole blood samples is more preferable, than checking notes in handling protocols. However, to date, only a few studies aimed to identify biomarkers reflecting delayed processing of whole blood [11, 13, 17, 47]. The applicability, on the other hand, is limited to some extent, because either a complex and difficult to analyze pattern is suggested [17], or the described tool contains biomarkers like lactate [13, 47], which could be greatly affected in their blood concentration by physiological as well as disease states. Liu X. and colleagues identified and rigorously validated in more than 2000 serum and plasma samples (4E, 14Z)-sphingadienine-C18-1-phosphate (common name: sphingosine-1-phosphate d18:2; S1P-d18:2) as a biomarker to verify compliance with SOP-prescribed time for processing into plasma or serum and/or time-to-storage of whole blood at 4 °C, i.e., to identify occasional delays, accidental errors, or systematic deviations in the handling of whole blood [11]. Cutoff levels for S1P-d18:2 (plasma: ≤ 0.085 μg/ml; serum: ≤ 0.154 μg/ml) are also given, allowing (bio)analytical scientists to decide whether or not sample quality is acceptable for the intended use, even without any knowledge of the whole blood handling performed from collection to centrifugation. Noteworthy, S1P is a class of lipids well known to show alterations in their blood levels due to physiological conditions or diseases states [48–50], but Liu et al. showed that alterations caused by these conditions are minor in comparison to changes based on pre-analytical errors [11], i.e., far below the S1P-d18:2 cutoff levels for the separation of plasma and serum samples of good and less suited quality for metabolomics and lipidomics studies.

### Freezing and long-term storage of plasma and serum

Usually, metabolomics and lipidomics analyses are not performed at the same day as body fluids’ collection. Hence, the samples need to be stored frozen until further analyses. The ideal way to freeze samples, like stepwise freezing, snap freezing, etc., and whether different freezing strategies show any effect on the metabolome is still unclear. Usually, samples are stored in aliquot days, weeks, or years in laboratory freezers or biobanks under standardized conditions at −80 °C or below. However, at the sites of body fluid collection, i.e., in a hospital or study ward, −80 °C freezers do usually not exist nearby. Therefore, it is not uncommon to use −20 °C freezers as intermediate storage for body fluids either for some hours, but also for days until the next set of samples is transported to the site of long-term storage at −80 °C or below. This raises the question about the stability of metabolite and lipid pattern in body fluids under different storage conditions.

In the context of sample storage, effects of freeze-and-thaw cycles are also of interest, because in every biomedical study, the number of stored aliquots is limited. Thawing, and as time goes on, refreezing of limited valuable sample material is unavoidable. Moreover, it should be considered that the sample stability is also highly dependent on the frequency and duration of opening the door of the freezer (e.g., in a hospital ward), which is not only true for storage at −20 °C but also at −80 °C. However, thawing of samples standing close to the open door of freezer occurs much faster at −20 °C than at −80 °C. Special attention should be whether self-defrosting −20 °C freezers are in use.

#### Storage at −20 °C

There are very few reports on the stability of samples at −20 °C intended for metabolomics and lipidomics investigations exist, because this is not the common storage temperature. At −20 °C, residual enzymatic activity cannot be excluded [20]. Additionally, polyunsaturated fatty acids (PUFA) are sensitive to degradation when stored at −20 °C [20, 51, 52]. A panel of 133 oxylipins was stable for up to 5 days at −20 °C in EDTA plasma [14]. After 4 weeks of storage, PUFAs were highly degraded and this effect was volume dependent, i.e., lower in 250 μl than in 80 μl [51]. On the other hand, a non-targeted metabolomics approach that included 2600 traits revealed only minor changes of the plasma metabolome after storage at −20 °C for 4 weeks, but this study was performed with only 4 different samples [53]. Even in organic solution, i.e., independent of possible effects of compounds included in body fluids, a standard mixture of 28 hydrophilic endogenous compounds showed stability for only 2 weeks at −20 °C [54]. To conclude, storage of lipids at −20 °C can be critical even for a short period of time. Short-term storage of body fluids at −20 °C for a few days before long-term storage at −80 °C or lower temperatures for metabolomics analysis could be feasible, but preliminary stability tests are recommended.

#### Storage at −80 °C and below

Storage at a temperature of −80 °C for months and years is most common for body fluids. At a minimum of 15 months, total plasma oxylipins are robust in EDTA plasma stored at −80 °C [14, 20]. The authors recommended adding the free radical scavenger butylated hydroxytoluene (BHT) to the samples directly after plasma separation [14]. After 2.5 years of storage at −80 °C, negligible changes were observed for amino acids, LPCs, and PCs in lithium-heparin plasma from 21 non-fasted individuals in an NMR-driven metabolomics study [44]. In a sophisticated targeted mass spectrometric study, Haid et al. investigated the storage stability of a profile of 111 metabolites in EDTA plasma over 5 years at −80 °C [55]. In contrast to the less sensitive NMR study, Haid et al. also covered low abundant plasma metabolites. After 5 years of storage at −80 °C, no significant changes were detected for half of all metabolites, and the rest showed minor alterations of ≤ 15% [55], i.e., far below inter-individual differences in biomedical metabolite profiling. The approach included metabolite classes of amino acids, glycerophospholipids, sphingolipids, hexoses, and acylcarnitines. In EDTA plasma stored for up to 16 years, Wagner-Golbs et al. revealed, using NMR profiling of 231 metabolites, only 2% changes in the first 7 years of storage at −80 °C [56]. After 16 years, the levels of 26% of all metabolites were significantly altered in comparison to latest samples of the same individuals; mainly affected were complex lipids, fatty acids, metabolites from energy metabolism, and amino acids [56]. However, the authors mentioned as a limitation of their study that due to the study design, they cannot exclude that changes in lifestyle, diseases, etc., of their participants during these 16 years, which may have affected and biased their findings and conclusions [56]. In addition to the ideal storage of deep-frozen samples at −196 °C (liquid nitrogen), Haid and colleagues demonstrated and recommended a strategy to compensate for storage effects at −80 °C on metabolite levels by a regression model [55]. A prerequisite for the application of this model is the storage of sufficient number of aliquots of either pooled study samples or comparable samples together with the study samples to allow repetitive measurements of these pooled QC sample aliquots over the whole storage time period to generate the regression model [55].

To conclude, slight changes during long-term storage at −80 °C cannot be prevented, but the changes are not pronounced and can be corrected by sophisticated strategies such as the regression model mentioned above. The data also illustrate that analysis of samples with different storage ages may significantly affect the final profiling results, at least for some metabolites. On the other hand, sample collection in complex human biomedical projects and multi-center studies usually takes months or even years. Hence, it is often unavoidable to mix samples of different storage age when metabolite or lipid profiling of a large cohort is performed. For the interpretation of those data, in addition to the variability due to different storage times (e.g., ±15% [55]), it is important to consider that the biological and inter-individual variability is usually much higher. This assumption is supported by numerous publications reporting valid data achieved in sets of body fluids stored for various periods at −80°C.

#### Effects of unavoidable repetitive freezing and thawing of serum or plasma aliquots

In most biomedical studies, long-term storage of many aliquots of body fluids is common practice allowing to perform various analysis. However, the number of these aliquots is continuously decreasing over time, particularly for the most interesting samples. This inevitable leads to repetitive thawing and refreezing to keep all residues of the limited sample material. As a consequence, the composition of the metabolome or lipidome of these refrozen aliquots could be affected.

How often serum or plasma samples need to be repeatedly thawed is highly dependent on the number of aliquots stored. Before the study begins, this issue, as well as the volume of each aliquot, should be carefully considered, thereby respecting the storage space and practical feasibility of aliquot generation (Tables 1 and 2). Choosing a smaller number of aliquots usually results in higher volumes and consequently more leftovers to be refrozen.

Variations in room temperature, exposure time to room or other temperatures, activation of enzymes inside the sample, etc., may occur from day to day during thawing. Since thawing at room or even higher temperature (hand warmth) is very fast, it is regularly applied. Four thawing cycles for 30 min at room temperature followed by refreezing at −80°C showed significant changes of serum and plasma levels of phospholipids, glycerol, and other lipids, as well as carnitine, choline, proline, acetone, glucose, alanine, and pyruvate in two independent NMR-driven studies [44, 57]. In contrast, four-times thawing EDTA plasma at 4 °C (iced water) and refreezing at −80 °C led only to marginal alterations in the EDTA plasma metabolome, which was studied using a non-targeted LC-MS approach [7]. Several years later, these investigations were repeated and the findings were confirmed by Goodman et al. [58]. Metabolites from lipid metabolism and central carbon metabolism, as well as antioxidants and nucleotides, were among the few compounds that were sensitive to repeated three times refreezing [58]. Helmschrodt et al. investigated the sensitivity of some reactive oxygen species-derived oxysterols to repetitive thaw cycles and found that up to nine cycles had no effect [59]. A mixture of 11 polar standard compounds resisted to 10 freeze-thaw cycles [54]. Noteworthy, volatile metabolites should be absolutely excluded from repetitive freeze-thaw cycles due to evaporation [44, 57].

Interestingly, in a PCA score-plot, individual differences in the sensitivity to freeze-thaw cycles of the EDTA plasma metabolome were obvious for two out of ten study participants [7]. Similar individual effects had been reported in an NMR-driven study [44]. The authors of this study speculated that a high content of lipids could be the reason for these differences in individual plasma samples.

In general, it is recommended to thaw body fluids < 10 °C for metabolomics and lipidomics analyses to prevent significant changes of the metabolite or lipid profiles [7, 58, 60]. The unavoidable freeze-and-thaw cycles should be carefully documented and a mixing of aliquots should be avoided, at least for metabolomics and lipidomics analysis.

## Urine

Urine, in contrast to blood, is an easy and non-invasive to collect body fluid, which is also well suited for metabolomics analysis, as documented by a considerable number of publications (Fig. 2). Normal urine is a transparent, yellowish, almost cell-free body fluid with very low protein content. The risk of alterations of the metabolome after collection, i.e., during transportation, handling, and processing is very low risk. A totally different situation exists in disease states. The composition of urine can change significantly (Fig. 3B), and carries a high risk for pre-analytical changes of the metabolome [16]. Hence, from the pre-analytical point of view, urine samples in biomedical studies may also contain enzymes, cellular, as well as other compounds (Fig. 3C), which is in clear contrast to urine from healthy donors. This means, urine samples can be highly contaminated with metabolic active compounds of microbiological and human origin or be almost free of any contaminations. Consequently, data from high-resolution metabolomics investigations could be affected to various extents based on pre-analytical issues during sample collection or transportation.

Urine test strips are an easy and very fast to perform option to assess this risk. Further on, they are well suited to achieve very useful qualitative information about the composition of urine (including possible compounds affecting the metabolome) and the health state of an individual. Urine test strips can be applied directly after collection and before any further sample processing qualitative information of the number of leukocytes, erythrocytes, increased protein content, fasted state (ketone bodies), as well as pH value, and many others are included in the tests on this strip. Noteworthy, the pH values of urines, in contrast to blood, can vary quite a lot between individuals. But to date, no data about possible pH-dependent effects on the metabolome and prevention strategies by buffering are available. Monitoring urine pH during collection is, to the best of our knowledge, only used in routine HCl-acidified urine analysis of catecholamines and some other compounds, but not in biomedical metabolomics projects.

Other than previously mentioned limitations, various types of urine samples need special considerations with respect to pre-analytical issues, which will be discussed in the following chapters.

### Urine sampling need pre-tests to circumvent analytical limitations

Urine samples, whether they are spot urine or 24-h collection urine, are collected in containers of different size made from plastic, and the same is true for urine collected in drainage bags from in-patients. Pediatric and newborn samples are collected in special soft plastic bags with hypoallergenic skin adhesive. As mentioned above (Collection tubes may introduce chemical noise), the type and quality of plastic used by various brands can differ. Therefore, two major pre-analytical pitfalls should be checked before the start of a study. The first is the adsorption of urinary metabolites to the plastic surface, in particular during 24-h urine or drainage bag collection (both associated with a rather long exposure time to the plastic surface), and the second, the release of interfering compounds like plasticizer into the sample. Furthermore, it is not uncommon to clean and reuse 24-h urine collection containers, so the risk of remaining detergents and subsequent contamination of the next urine sample should be considered.

### Urine specimen types and their strengths and limitations

Urine is collected in the bladder before excretion. As a consequence, the type of the analyzed urine specimen leads to differences in the composition of the urinary metabolite profile of an individual. The types other than spot urine, which is collected randomly, are first morning urine (MU), second MU, 24-h collection urine, and drainage bag urine as a special kind of collection urine. Each type has its specific characteristics comprising pros and cons for metabolomics analyses, as well as the need for specific pre-analytical considerations. For spot urine, which is collected randomly, as well as for first and second morning urine, it is imperative to collect midstream urine. Otherwise, a mass of metabolic active epithelial cells from the genital surface will reach the urine sample tube, even when collected from healthy donors, the urine will be contaminated (Fig. 3C).

Midstream first MU is the urine excreted directly after getting up. It contains all metabolites collected in the bladder overnight. Therefore, the metabolite pattern in first MU may also reflect nutrition on the previous day, particularly of the evening [61]. It can be seen as a kind of collection urine.

Second MU, usually collected still in the fasted state between 7 and 10 am, stands for the next excretion after first MU. In the metabolome of this type of specimen, the nutrition effects of previous day are greatly reduced [61]. Given that the study subjects are well-instructed midstream, second MU is a more standardized sample material than the first MU. Normalized to urinary creatinine concentration, it is also the most common sample material used for quantitative analysis in Clinical Chemistry. But second MU is not the sample material of choice for quantitative clinical routine analysis, which is 24-h collection urine.

The collection container for 24-h urine should be of adequate size, usually 3 l or more, as well as allowing UV-light protection of the sample. The concentration measured in 24-h collection period and in drainage bag urines can be directly related to the volume of urine collected. Consequently, the quantification is more reliable than the creatinine-normalized quantification in second MU, assuming that there is no collection error by the patient. Hence, collection urine would also be most suitable for metabolomics analysis. However, apart from several advantages, there are also disadvantages that argue against the use of collection urine for metabolomics (see also Cooling during collection and transportation preserves the metabolome). Effects and variabilities on the metabolite pattern caused by food intake are unavoidable and urinary markers for milk, cheese, meat, fish, and other nutrition have been described in metabolomics studies [62–64]. For 24-h collection urine, the study participant or patient is responsible for not missing any urine volume and for keeping the urine collector cool during this long period. Overall, the 24-h collection period is cumbersome and error-prone. Four most common errors are (a) not emptying the bladder before starting the collection period, (b) not emptying the bladder before the end of the 24-h collection period, (c) forgetting to collect one or more urine voids, and (d) forgetting to cool the container during the collection period. Other potential problems can be caused by detergents remaining in the collection container after cleaning, and by the patient, e.g., estimating the volume of a forgotten urine fraction and adding the respective volume of water into the 24-h collection container. For urines collected in drainage bags, cooling during the period of sampling is almost impossible, which entail for this sample material the limitation to use only cell-free samples for metabolomics investigations.

In general, all types of urine samples have their strengths and weaknesses. For metabolomics studies, I would recommend midstream second MU collected after an overnight fast (until sample collection, only drinking of water is allowed) (Table 2). Based on the direct and possibly strong effects of the diet on the previous day in an ideal study 20 h before sample collection, a standardized vegetarian diet should be started to harmonize the diet effect in the study group [61], which, however, is hardly feasible in daily practice.

### Cooling during collection and transportation preserves the metabolome

The collection of urine is not only performed in the clinic but also at home. In both situations, as well as during longer sample transportation, temperature is a relevant pre-analytical factor. Rotter et al. investigated, using a targeted approach covering 63 metabolites, the stability of this metabolite profile in pooled morning urine of 6 healthy female individuals under various storage conditions between 2 and 24 h [65]. At room temperature (20 °C), no change in the concentration of 49 metabolites within 24 h was observed. However, a decrease of 30 to 60% was detected for some metabolites, namely arginine, methionine, serine valine, leucine/isoleucine, and hexose. Cooling with a cold pack for one day (9 °C) still showed 40% decrease of branched-chain amino acids as well as arginine, and an increase of C6:1-carnitine by 16% [65]. In an NMR-driven study investigating non-pooled spot urine samples from eleven healthy individuals, Budde et al. reported only minor, non-relevant changes in terms of inter-individual differences after a 24-h exposure at 10 °C, and no changes within 10-h exposure [66]. Other metabolomics studies confirmed only negligible alterations in urine samples after 24 h at 4 °C compared to the observed inter-individual variabilities [30, 67, 68]. In pooled urines, even after 72 h at 4 °C, alterations in the metabolome were very small [69]. When tested at room temperature, Roux et al. demonstrated after 12 h significant changes in 7% of the 280 metabolites in pooled urines in a non-targeted approach [69]. Before drawing general conclusions from the described findings, it is important to note that only urines from healthy individuals were studied but not urines containing enzymes, cellular, as well as other compounds (see Fig. 3C). Furthermore, the enclosed metabolite profiles were quite different.

An alternative strategy to cooling is the addition of stabilizers such as borate or thymol into the sample container to reduce or inhibit the growth of bacteria and fungi, as well as metabolic activity of other cells [70]. To the best of our knowledge, however, this strategy is not widely used (at least in hospitals for diagnostic routine purposes). Wang et al. reported effective stabilization of a metabolite profile of 158 urinary metabolites by thymol, while the addition of boric acid caused alterations of the metabolome (n = 20 participants) detected by LC-MS [71]. Additionally, Lauridsen et al. tested other inhibitors and demonstrated that the use of 0.1% sodium azide is not critical for NMR spectra; however, 1% sodium fluoride led to shifts [67].

In summary, during the 24-h urine collection period, as well as during transportation of any type of urine, it is preferable to keep samples at 4 °C to largely reduce enzyme activities, cellular metabolism in general, and microbiological growth. The addition of bacteriostatic agents to urine collectors as well as pH adjustment before collection is not common and should be verified before application.

### Freezing and storage of urine

Space for long-term sample storage is always limited. Consequently, at least in clinical area, there is still debate on whether urine can be stored at −20 °C in a less costly manner. In a comparison of metabolite profiles in urine samples frozen immediately after collection, no difference in NMR spectra was observed between storage at −25 and −80 °C after 26 weeks [67]. In a sophisticated LC-MS approach applying reversed-phase and hydrophilic interaction chromatography, only minor differences in a pattern of more than 5000 features were detected in bovine urine stored at −20 °C or −80 °C for 144 days [72]. Ultrafiltration with a cutoff filter of 10 kDa was performed before storage; hence, the pooled bovine urine samples were absolutely free from cells and almost free from proteins [72]. Ultrafiltration of urine could be an efficient but time-consuming and expensive way to stabilize urinary metabolomes.

Freeze-drying of urine and subsequent storage at −20 °C or −80 °C is another option to preserve sample quality [72]. At −20 °C, the metabolite profile of > 5000 features in freeze-dried samples was nearly stable over the 144 days studied [72]. At −80 °C, no difference was observed between freeze-dried and non-freeze-dried samples [72]. When using freeze-dried samples, care should be taken during resuspension of metabolites before high-resolution analysis.

To sum up, a weakness of pre-analytical reports is that all stability studies were performed exclusively on urines from healthy individuals. This limits the general applicability of the data obtained to all types of biomedical studies. It should be considered that depending on disease states or even improper sampling of midstream morning urine from healthy subjects, various cellular and enzymatic active compounds may be present in the urine (see Fig. 3C). This might alter the metabolome during collection period, transportation, or even after storage. In addition, some of the perfect conditions described for preserving the urine metabolome, like immediate urine freezing [67] or ultrafiltration [72], are difficult to apply in common biomedical studies conducted outside a research institute in a clinical surrounding. To be on the safe side, pre-tests adapted to the individual situation of each biomedical study are advisable before long-term storage of urine.

## Cerebrospinal fluid (CSF)

Although the sample material is not easy to collect, CSF is of great interest for (bio)analytical chemists to study (patho)physiology of the central nervous system and to identify diagnostic biomarkers through metabolomics and lipidomics analysis, which is reflected in numerous reports (Fig. 2). The accessibility for sampling CSF via the spinal canal is much more challenging for physicians than blood sampling. An accidental admixture of blood due to injury of a capillary during puncture occurs regularly. Normal CSF is colorless, water-clear, with almost no cells (< 5 cells per μl), and the protein concentration is very low (below 500 mg/l) [25]. In simple terms, CSF is an ultrafiltrate of blood and the filter is the blood-brain barrier (BBB). However, the BBB is much more than a filter; it is a selective barrier. It allows only diffusion of water and various hydrophobic compounds and performs selective transportation of other molecules needed for the function of the brain. Various disease states lead to alterations in the function and permeability of the BBB. The most common are infectious/inflammatory diseases. In these situations, the cell count in CSF can change dramatically, increasing up to several thousand leukocytes/μl. Metabolically active bacteria, yeasts, tumor cells, erythrocytes, leukocytes, and some other cells can be also found in CSF samples under pathological conditions. These cells may alter the CSF metabolome after sample collection, depending on the type of cells, their number, and their metabolic activity.

For routine diagnostics as well as for metabolomics/lipidomics investigations of CSF, it is mandatory to collect CSF and blood in pairs at the same time. International guidelines recommend centrifugation of CSF (2000×*g* at 4 °C) and blood within 2 h after collection [73]. Collection of pairs is an essential requirement, because alterations in the concentration of metabolites in CSF may occur due to changes in blood in the systemic circulation without pathophysiological changes in the central nervous system. By calculating CSF/serum or CSF/plasma quotients, misinterpretation of metabolomics and lipidomics data caused by such situations can be avoided.

Although normal CSF is like an ultrafiltrate from blood, pre-analytical considerations, particularly when studying pathological liquor samples, are important. Haijes et al. investigated recently various aspects that may alter the CSF metabolome after collection [74]. Centrifuged individual CSF biobank samples from a dementia cohort were pooled and analyzed for > 1800 features by direct-infusion MS. They found that storage of centrifuged (i.e., cell-free) CSF at room temperature (18–22 °C) for up to 8 h is acceptable [74]. An increased number of leukocytes in pathological CSF lead to alterations of metabolite and protein levels in uncentrifuged samples already after 30 min at room temperature [75]. Cooling of centrifuged CSF below 8 °C for up to 72 h showed only minor effects on metabolite levels, as did storage at −20 °C for 2 months [74]. In a profile of 36 amines, the levels of 31 were stable for up to 2 h when refrigerated immediately after CSF collection [76]. Furthermore, up to three freeze-and-thaw cycles of centrifuged CSF resulted in a relatively unaffected metabolome [74]. Regarding patient preparation, Saito et al. reported recently that postprandial state had lower impact on 130 hydrophilic metabolites and 340 lipids in CSF of nine healthy subjects than inter-individual variations [77]. Less than 10% of CSF metabolites show significant changes. This led the authors to conclude that metabolites and lipids in CSF are not directly associated with food intake [77].

In summary, cooling and timely centrifugation is the easiest, but most effective way to stabilize the metabolome and lipidome of CSF (Table 2). Once the cellular compounds are removed, CSF is quite stable, even for a longer period of time, even at room temperature. However, based on the pathological state of a patient, such as cerebral hemorrhage or dysfunction of the BBB, a high variability in the sample composition associated with potential negative pre-analytical effects on metabolomes is possible and should be respected.

## Study participants

### Preparation of sophisticated questionnaires and instructions to avoid pre-analytical shortcomings

Outliers or high variations of metabolomics and lipidomics results in biomedical profiling projects are a challenge for data evaluation and interpretation. Many of them could easily be prevented as they occur before the collection of samples. Common causes are either vague or incomplete instructions of study participants about correct preparation in the period before sample collection or misunderstandings between instructor and study participant.

Clear and comprehensive guidelines and checklists for the instruction of study participants, which are jointly prepared by all involved scientists, can help to avoid or minimize outliers and subsequent large data variabilities (Table 1). Additionally, comprehensive but not excessive questionnaires may also be useful in this context. For instance, a look in the corresponding questionnaire may explain detected outliers and can also provide reasonable justification to exclude outlier data from further evaluation.

Central question for creating a guide for metabolome and lipidomics study participants or questionnaire is simply what factors or activities can influence the metabolome and lipidome in body fluids or tissues. Such factors that affect metabolome can be divided in two sections, uninfluenceable ones like sex [78–80], age [78–80], and ethnicity [81, 82], and influenceable ones such as the time of day of sample collection [83–86], physical activity, pre-/postprandial state [87]. Table 1 provides a summary of suggested general points that should be considered. Three aspects could represent important pitfalls and are discussed in the following paragraphs.

Aspect #1 (questionnaire): Individual needs and preferences of study subjects are manifold and can significantly alter the metabolome and lipidome. Therefore, this should be carefully asked and documented, as for example, dietary supplements (vitamins, lipids (fish oil), iron, etc.), drugs, special or trendy diets (gluten-free, vegan, paleo, etc.), smoking, daily amount of tea or coffee, and alcohol consumption (Table 1). Particularly, detailed questions about dietary supplements need to be included because they are usually not perceived as a kind of “medication” affecting human metabolism. This means that supplement intake is usually not reported when study subjects are asked only about medication intake. Yet, fish oil capsules or flavonoid-rich supplement as an example may heavily affect distinct metabolites/lipids, or modify metabolic pathways as well as the inflammatory status and other mechanisms in the body [88, 89]. The same is likely to be true for minerals, plant extracts, vitamin pills, amino acid or protein shakes, and many others.

In general, it is quite difficult to find participants/patients who do not take anything, i.e., at younger age, people consume regularly mainly food supplements and > 45 years of age medications (often in combinations with supplements). Consequently, it is almost impossible to assemble a study cohort or select samples from a biobank with metabolomes and lipidomes free of any xenobiotic “side effects.” Therefore, informative entries in questionnaires accessible for (bio)analytical chemists are highly relevant.

Aspect #2 (participant instruction): The vast majority of biomedical metabolomics or lipidomics studies prefer the analysis of samples collected in the fasted state (Table 1). Hence, a clear instruction of study participants about fasting prior sample collection is important. A common fasting period is usually not less than 12 h, which can be most easily achieved by overnight fasting. “Fasting” is usually understood as “[...] not eating for a defined period of time […],” but drinking is allowed. What kind of beverage is allowed in the morning? It should be considered that people are often unaware that drinking only one cup of milk-sweetened coffee, tea, or sweetened beverage after getting up in the morning on the day of the study changes the composition of the metabolome [29, 90]. Drinking only pure water without any additives, like artificial flavors or others, in the morning is rather uncommon but the only acceptable drink before collection of body fluids in the fasted state. Pure tea or coffee should also be excluded [90–92].

Subjects should also be instructed to avoid all kinds of extreme and unaccustomed metabolic and physiological challenges (e.g., stress (being late), unusual physical activity, prolonged starvation, unaccustomed exercise, or food excesses) on the day or days before sample collection.

Aspect #3 (scheduling): The time of day of sample collection is another factor that may have major effects on the metabolome or lipidome of interest (Table 1). Not only diet and nutritional state [93, 94], but also circadian effects on the metabolome in mice and humans [83–85] and the human lipidome [86] should be taken into consideration. About 13% of studied human plasma lipidome exhibited partially opposed circadian rhythmicity, most pronounced for the classes of phosphatidylcholines, diacylglycerols, and triacylglycerols [86]. The group of H.R. Ueda reported the quantification of circadian oscillating metabolites in human and mouse blood and in several mouse organs [83–85]. The human study was strictly controlled for confounding effects of activity level, light, temperature, sleep, and food intake [84]. Consequently, the time point of sample collection should be kept consistent within a study (Table 1). Circadian oscillation should also be kept in mind as a cause for discrepancies between findings from different sites in multi-center studies or from studies in different literatures.

Usually, it is not a problem to perform the sample collection in the morning, ideally between 7 and 10 a.m. after a 12-h fasting period. But, in studies recruiting tens of thousands of subjects at different sites, it is quite difficult to collect all samples at the same-day time due to logistical reasons. In such multi-center studies, like national cohort studies, accurate documentation of the time of sample collection and the metabolic status of the participant is essential. Samples collected post-prandial and/or not between 7 and 10 a.m. should be excluded from metabolomics and lipidomics investigations; otherwise, outliers and high variability in the data are guaranteed.

### Apparently healthy, asymptomatic individuals challenge the compilation of healthy control groups

A comparison of the achieved disease-related lipid or metabolite profile with a healthy control group is one of the first and also decision-making steps in most biomedical targeted and non-targeted biomarker studies in animals and humans. It is usually the basis for discussing whether or not to proceed with further profiling investigations. However, the compilation of a healthy cohort can be quite challenging since asymptomatic, apparently healthy individuals need to be identified and excluded. Otherwise, there is a very high risk for the detection of misleading biomarker instead of disease-specific markers [95]. What might cause this to happen?

In humans, many diseases remain undiagnosed for years because the affected individuals are largely asymptomatic; i.e., they feel and seem to be apparently healthy. Examples include kidney and liver diseases, but also the widespread metabolic disease of type 2 diabetes with more than 430,000,000 affected subjects worldwide (half of them is not aware of having diabetes). A hallmark of many asymptomatic chronic diseases, as well as mild infectious diseases, is low-grade inflammation. In such cases, blood levels of the most common and sensitive inflammation marker C-reactive protein (CRP) are already slightly above the reference range. Ideally, CRP should be measured by a high-sensitive CRP assay in plasma or serum (known as hsCRP).

Undiagnosed, largely asymptomatic diseases can significantly alter the metabolome and lipidome, as recently shown in an elegant study by Pietzner et al. [95]. The authors examined urine and plasma samples from more than 900 apparently healthy individuals with slightly altered routine markers of inflammation in blood (CRP, white blood cell count, fibrinogen). Subjects with a CRP > 10 mg/l, reflecting acute inflammation, were excluded from the study. In a pattern of around 600 metabolites in each specimen, a significant association of inflammatory state was detected with almost 12% of all plasma metabolites and 4% in spot urine [95]. Consequences of the findings of Pietzner et al are (a) unnoticed, low-grade inflammation in apparently healthy humans can bias the metabolome in blood and urine; (b) the described CRP-associated metabolites could be suitable tools to identify subjects with subclinical inflammation in metabolomics data; and (c) the detection of these “inflammatory biomarkers” in another context in human metabolomics project can be a hint to be cautious in data interpretation, particularly to title these metabolites as specific biomarkers for particular diseases. In the worst case, uncontrolled health conditions may lead to a control group dominated by apparently healthy humans with low-grade inflammation (or other asymptomatic diseases). This can result in the discovery of biomarkers reflecting different stages of inflammation, rather than the intended specific markers related to the actual research question of the project.

The simplest but already very informative way to obtain several information on the health status of each study participant are the above-described urine test strips (see also introduction to Urine), ideally combined with the determination of hsCRP in blood. More detailed, but still easily obtained, are other common organ-specific blood tests, like creatinine for kidney function, gamma glutamyl transferase (γ-GT) for liver, and thyroid-stimulating hormone (TSH) for thyroid function. Ideal records about the individual behind each sample would contain a standard routine laboratory profile combined with data from a medical checkup, which is, however, time-consuming as well as cost-intensive. Therefore, these profiles are not widely used. Whenever possible, (bio)analytical chemists should have access to the anonymized health state data in the phase before sample preparation for high-resolution analyses begins. This information could be used either to verify the suitability of the sample set selected by a medical doctor or a biobank, or to self-select samples that are best suited to the intention of the respective metabolomics project.

To conclude, the phase before sample collection, which compromises the instruction of study participants, their habits, and recent activities, can bias the metabolome and lipidome and thus the outcome of a profiling study before a sample is even collected, prepared, or analyzed. Strong differences in metabolism and therefore in the metabolome can be expected based on time of day of sample collection, stress, pre- or postprandial state, intake of dietary supplements, unaccustomed physical activity before sample collection, life style factors (e.g., caffeine, alcohol, smoking), trendy diets, xenobiotics, drugs (see Table 1). The availability of routine laboratory records, completed questionnaires, etc., as basic information of all study participants is desirable.

## Conclusions

The quality of biomedical samples is of enormous importance for reliable and conclusive findings in lipidomics and metabolomics studies. The most critical steps that affect sample quality are identified to occur not in the laboratory of (bio)analytical chemists, but in the pre-analytical phase before sample collection, when metabolically active cells in blood, urine, or CSF may alter the lipidome or metabolome ex vivo. This means, crucial for good sample quality are the apparently simple steps from collection of body fluid to storage of the aliquots in the freezer. Since all these steps are well known (as well as possible pitfalls), the generation of generally applicable SOPs is possible. However, each sample collection and sample handling site in a hospital or a multi-center study has its own characteristics and limitations in terms of perfect sample processing. Therefore, one of the biggest challenges for (bio)analytical chemists to achieve the best possible sample quality is to find a balance between demands for perfect sample quality and the capabilities at the site of sample collection. This always involves compromises regarding perfect sample quality. But it is the important base for the generation of feasible SOPs that enable highly reproducible sample collection and handling at all sites (for suggestions, see Table 2), and subsequent successful, valid high-resolution metabolomics and lipidomics investigations.

Unfortunately, a generally applied and accepted strategy to verify and document the sample quality in biomedical profiling projects by quantifying quality biomarkers in random samples is still missing. In the future, it would be desirable to agree on biomarkers for the assessment of body fluid quality in high-resolution metabolomics and lipidomics approaches, and to document the quality grade, e.g., in the supplement of publications, as it is already a well-established standard for the analytical accuracy.

## References

[1] Carraro P, Zago T, Plebani M (2012). Exploring the initial steps of the testing process: frequency and nature of pre-preanalytic errors. Clin Chem.

[2] Lippi G, Guidi GC, Mattiuzzi C, Plebani M (2006). Preanalytical variability: the dark side of the moon in laboratory testing. Clin Chem Lab Med.

[3] Szecsi PB, Odum L (2009). Error tracking in a clinical biochemistry laboratory. Clin Chem Lab Med.

[4] Züllig T, Trötzmüller M, Köfeler HC (2020). Lipidomics from sample preparation to data analysis: a primer. Anal Bioanal Chem.

[5] Vuckovic D (2012). Current trends and challenges in sample preparation for global metabolomics using liquid chromatography-mass spectrometry. Anal Bioanal Chem.

[6] Dunn WB, Broadhurst D, Begley P, Zelena E, Francis-McIntyre S, Anderson N (2011). Procedures for large-scale metabolic profiling of serum and plasma using gas chromatography and liquid chromatography coupled to mass spectrometry. Nat Protoc.

[7] Yin P, Peter A, Franken H, Zhao X, Neukamm SS, Rosenbaum L (2013). Preanalytical aspects and sample quality assessment in metabolomics studies of human blood. Clin Chem.

[8] Paglia G, Del Greco FM, Sigurdsson BB, Rainer J, Volani C, Hicks AA (2018). Influence of collection tubes during quantitative targeted metabolomics studies in human blood samples. Clinica chimica acta; international journal of clinical chemistry.

[9] Allard L, Bowen RAR (2021). Preanalytical error: improper gel barrier formation in a serum separator tube despite appropriate centrifugation condition. Clinica chimica acta; international journal of clinical chemistry.

[10] Bowen RA, Remaley AT (2014). Interferences from blood collection tube components on clinical chemistry assays. Biochemia Medica.

[11] Liu X, Hoene M, Yin P, Fritsche L, Plomgaard P, Hansen JS (2018). Quality control of serum and plasma by quantification of (4E,14Z)-sphingadienine-C18-1-phosphate uncovers common preanalytical errors during handling of whole blood. Clin Chem.

[12] Wang Y, Carter BD, Gapstur SM, McCullough ML, Gaudet MM, Stevens VL (2018). Reproducibility of non-fasting plasma metabolomics measurements across processing delays. Metabolomics..

[13] Jain M, Kennedy AD, Elsea SH, Miller MJ (2017). Analytes related to erythrocyte metabolism are reliable biomarkers for preanalytical error due to delayed plasma processing in metabolomics studies. Clinica chimica acta; international journal of clinical chemistry.

[14] Koch E, Mainka M, Dalle C, Ostermann AI, Rund KM, Kutzner L (2020). Stability of oxylipins during plasma generation and long-term storage. Talanta..

[15] Liu X, Hoene M, Xiaolin W, Yin P, Häring HU, Xu GW (2018). Serum or plasma, what is the difference? Investigations to facilitate the sample material selection decision making process for metabolomics studies and beyond. Anal Chim Acta.

[16] Kirwan JA, Brennan L, Broadhurst D, Fiehn O, Cascante M, Dunn WB (2018). Preanalytical processing and biobanking procedures of biological samples for metabolomics research: a white paper, community perspective (for “Precision Medicine and Pharmacometabolomics Task Group”-The Metabolomics Society Initiative). Clin Chem.

[17] Kamlage B, Maldonado SG, Bethan B, Peter E, Schmitz O, Liebenberg V (2014). Quality markers addressing preanalytical variations of blood and plasma processing identified by broad and targeted metabolite profiling. Clin Chem.

[18] Bernini P, Bertini I, Luchinat C, Nincheri P, Staderini S, Turano P (2011). Standard operating procedures for pre-analytical handling of blood and urine for metabolomic studies and biobanks. J Biomol NMR.

[19] Hahnefeld L, Gurke R, Thomas D, Schreiber Y, Schäfer SMG, Trautmann S (2020). Implementation of lipidomics in clinical routine: can fluoride/citrate blood sampling tubes improve preanalytical stability?. Talanta..

[20] Jonasdottir HS, Brouwers H, Toes REM, Ioan-Facsinay A, Giera M (2018). Effects of anticoagulants and storage conditions on clinical oxylipid levels in human plasma. Biochim Biophys Acta Mol Cell Biol Lipids.

[21] Ammerlaan W, Trezzi JP, Lescuyer P, Mathay C, Hiller K, Betsou F (2014). Method validation for preparing serum and plasma samples from human blood for downstream proteomic, metabolomic, and circulating nucleic acid-based applications. Biopreserv Biobank.

[22] Lippi G, Blanckaert N, Bonini P, Green S, Kitchen S, Palicka V (2008). Haemolysis: an overview of the leading cause of unsuitable specimens in clinical laboratories. Clin Chem Lab Med.

[23] Marques-Garcia F (2020). Methods for hemolysis interference study in laboratory medicine - a critical review. Ejifcc..

[24] Guder WG, Naraynan S, Wisser H, Zwata B (2003). Samples: from the patient to the laboratory.

[25] Lehmann R. Chapter 3 - pre-analytics in biomedical metabolomics. In: Adamski J, editor. Metabolomics for biomedical research: Academic Press; 2020. p. 33-56.

[26] Wedge DC, Allwood JW, Dunn W, Vaughan AA, Simpson K, Brown M (2011). Is serum or plasma more appropriate for intersubject comparisons in metabolomic studies? An assessment in patients with small-cell lung cancer. Anal Chem.

[27] Yu Z, Kastenmuller G, He Y, Belcredi P, Moller G, Prehn C (2011). Differences between human plasma and serum metabolite profiles. PLoS One.

[28] Wolrab D, Chocholoušková M, Jirásko R, Peterka O, Mužáková V, Študentová H (2020). Determination of one year stability of lipid plasma profile and comparison of blood collection tubes using UHPSFC/MS and HILIC-UHPLC/MS. Anal Chim Acta.

[29] Allard E, Backstrom D, Danielsson R, Sjoberg PJ, Bergquist J (2008). Comparing capillary electrophoresis-mass spectrometry fingerprints of urine samples obtained after intake of coffee, tea, or water. Anal Chem.

[30] Dunn WB, Broadhurst D, Ellis DI, Brown M, Halsall A, O’Hagan S (2008). A GC-TOF-MS study of the stability of serum and urine metabolomes during the UK Biobank sample collection and preparation protocols. Int J Epidemiol.

[31] Santos Ferreira DL, Maple HJ, Goodwin M, Brand JS, Yip V, Min JL, et al. The effect of pre-analytical conditions on blood metabolomics in epidemiological studies. Metabolites. 2019;9(4):64. 10.3390/metabo9040064 PMC6523923 30987180

[32] Barri T, Dragsted LO (2013). UPLC-ESI-QTOF/MS and multivariate data analysis for blood plasma and serum metabolomics: effect of experimental artefacts and anticoagulant. Anal Chim Acta.

[33] Teahan O, Gamble S, Holmes E, Waxman J, Nicholson JK, Bevan C (2006). Impact of analytical bias in metabonomic studies of human blood serum and plasma. Anal Chem.

[34] Lesche D, Geyer R, Lienhard D, Nakas CT, Diserens G, Vermathen P (2016). Does centrifugation matter? Centrifugal force and spinning time alter the plasma metabolome. Metabolomics..

[35] Moretti F, Birarelli M, Carducci C, Pontecorvi A, Antonozzi I (1990). Simultaneous high-performance liquid chromatographic determination of amino acids in a dried blood spot as a neonatal screening test. J Chromatogr.

[36] Drolet J, Tolstikov V, Williams BA, Greenwood BP, Hill C, Vishnudas VK, et al. Integrated metabolomics assessment of human dried blood spots and urine strips. Metabolites. 2017;7(3):35. 10.3390/metabo7030035 PMC5618320 28714878

[37] Palmer EA, Cooper HJ, Dunn WB (2019). Investigation of the 12-month stability of dried blood and urine spots applying untargeted UHPLC-MS metabolomic assays. Anal Chem.

[38] Strnadová KA, Holub M, Mühl A, Heinze G, Ratschmann R, Mascher H (2007). Long-term stability of amino acids and acylcarnitines in dried blood spots. Clin Chem.

[39] Fingerhut R, Ensenauer R, Röschinger W, Arnecke R, Olgemöller B, Roscher AA (2009). Stability of acylcarnitines and free carnitine in dried blood samples: implications for retrospective diagnosis of inborn errors of metabolism and neonatal screening for carnitine transporter deficiency. Anal Chem.

[40] Volani C, Caprioli G, Calderisi G, Sigurdsson BB, Rainer J, Gentilini I (2017). Pre-analytic evaluation of volumetric absorptive microsampling and integration in a mass spectrometry-based metabolomics workflow. Anal Bioanal Chem.

[41] Anton G, Wilson R, Yu ZH, Prehn C, Zukunft S, Adamski J (2015). Pre-analytical sample quality: metabolite ratios as an intrinsic marker for prolonged room temperature exposure of serum samples. PLoS One.

[42] Shurubor YI, Matson WR, Willett WC, Hankinson SE, Kristal BS (2007). Biological variability dominates and influences analytical variance in HPLC-ECD studies of the human plasma metabolome. BMC Clin Pathol.

[43] Yang W, Chen Y, Xi C, Zhang R, Song Y, Zhan Q, et al. LC-MS/MS-based plasma metabonomics delineate the effect of metabolites’ stability on reliability of potential biomarkers. Anal Chem. 2013;85(5):2606–10. 10.1021/ac303576b 23387999

[44] Pinto J, Domingues MR, Galhano E, Pita C, Almeida Mdo C, Carreira IM (2014). Human plasma stability during handling and storage: impact on NMR metabolomics. Analyst..

[45] Breier M, Wahl S, Prehn C, Fugmann M, Ferrari U, Weise M (2014). Targeted metabolomics identifies reliable and stable metabolites in human serum and plasma samples. PLoS One.

[46] Ghini V, Quaglio D, Luchinat C, Turano P (2019). NMR for sample quality assessment in metabolomics. New Biotechnol.

[47] Trezzi JP, Bulla A, Bellora C, Rose M, Lescuyer P, Kiehntopf M (2016). LacaScore: a novel plasma sample quality control tool based on ascorbic acid and lactic acid levels. Metabolomics..

[48] Baranowski M, Charmas M, Dlugolecka B, Gorski J (2011). Exercise increases plasma levels of sphingoid base-1 phosphates in humans. Acta Physiol (Oxford).

[49] Yatomi Y (2008). Plasma sphingosine 1-phosphate metabolism and analysis. Biochim Biophys Acta.

[50] Nixon GF (2009). Sphingolipids in inflammation: pathological implications and potential therapeutic targets. Br J Pharmacol.

[51] Pottala JV, Espeland MA, Polreis J, Robinson J, Harris WS (2012). Correcting the effects of -20 degrees C storage and aliquot size on erythrocyte fatty acid content in the Women's Health Initiative. Lipids..

[52] Metherel AH, Aristizabal Henao JJ, Stark KD (2013). EPA and DHA levels in whole blood decrease more rapidly when stored at -20 degrees C as compared with room temperature, 4 and -75 degrees C. Lipids..

[53] Gonzales GB, De Saeger S (2018). Elastic net regularized regression for time-series analysis of plasma metabolome stability under sub-optimal freezing condition. Sci Rep.

[54] Culp-Hill R, Reisz JA, Hansen KC, D’Alessandro A (2017). Investigation of the effects of storage and freezing on mixes of heavy-labeled metabolite and amino acid standards. Rapid Commun Mass Spectrom.

[55] Haid M, Muschet C, Wahl S, Romisch-Margl W, Prehn C, Moller G (2018). Long-term stability of human plasma metabolites during storage at -80 degrees C. J Proteome Res.

[56] Wagner-Golbs A, Neuber S, Kamlage B, Christiansen N, Bethan B, Rennefahrt U, et al. Effects of long-term storage at -80 °C on the human plasma metabolome. Metabolites. 2019;9(5):99. 10.3390/metabo9050099 PMC6572224 31108909

[57] Fliniaux O, Gaillard G, Lion A, Cailleu D, Mesnard F, Betsou F (2011). Influence of common preanalytical variations on the metabolic profile of serum samples in biobanks. J Biomol NMR.

[58] Goodman K, Mitchell M, Evans AM, Miller LAD, Ford L, Wittmann B (2021). Assessment of the effects of repeated freeze thawing and extended bench top processing of plasma samples using untargeted metabolomics. Metabolomics..

[59] Helmschrodt C, Becker S, Thiery J, Ceglarek U (2014). Preanalytical standardization for reactive oxygen species derived oxysterol analysis in human plasma by liquid chromatography-tandem mass spectrometry. Biochem Biophys Res Commun.

[60] Pizarro C, Arenzana-Rámila I, Pérez-del-Notario N, Pérez-Matute P, González-Sáiz JM (2016). Thawing as a critical pre-analytical step in the lipidomic profiling of plasma samples: new standardized protocol. Anal Chim Acta.

[61] Liu X, Yin P, Shao Y, Wang Z, Wang B, Lehmann R (2020). Which is the urine sample material of choice for metabolomics-driven biomarker studies?. Anal Chim Acta.

[62] Munger LH, Trimigno A, Picone G, Freiburghaus C, Pimentel G, Burton KJ (2017). Identification of urinary food intake biomarkers for milk, cheese, and soy-based drink by untargeted GC-MS and NMR in healthy humans. J Proteome Res.

[63] Khodorova NV, Rutledge DN, Oberli M, Mathiron D, Marcelo P, Benamouzig R (2019). Urinary metabolomics profiles associated to bovine meat ingestion in humans. Mol Nutr Food Res.

[64] Cheung W, Keski-Rahkonen P, Assi N, Ferrari P, Freisling H, Rinaldi S (2017). A metabolomic study of biomarkers of meat and fish intake. Am J Clin Nutr.

[65] Rotter M, Brandmaier S, Prehn C, Adam J, Rabstein S, Gawrych K (2017). Stability of targeted metabolite profiles of urine samples under different storage conditions. Metabolomics..

[66] Budde K, Gok ON, Pietzner M, Meisinger C, Leitzmann M, Nauck M (2016). Quality assurance in the pre-analytical phase of human urine samples by (1)H NMR spectroscopy. Arch Biochem Biophys.

[67] Lauridsen M, Hansen SH, Jaroszewski JW, Cornett C (2007). Human urine as test material in 1H NMR-based metabonomics: recommendations for sample preparation and storage. Anal Chem.

[68] Lenz EM, Bright J, Wilson ID, Morgan SR, Nash AF (2003). A 1H NMR-based metabonomic study of urine and plasma samples obtained from healthy human subjects. J Pharm Biomed Anal.

[69] Roux A, Thevenot EA, Seguin F, Olivier MF, Junot C (2015). Impact of collection conditions on the metabolite content of human urine samples as analyzed by liquid chromatography coupled to mass spectrometry and nuclear magnetic resonance spectroscopy. Metabolomics..

[70] Meers PD, Chow CK (1990). Bacteriostatic and bactericidal actions of boric acid against bacteria and fungi commonly found in urine. J Clin Pathol.

[71] Wang X, Gu H, Palma-Duran SA, Fierro A, Jasbi P, Shi X, et al. Influence of storage conditions and preservatives on metabolite fingerprints in urine. Metabolites. 2019;9(10):203. 10.3390/metabo9100203 PMC6836253 31569767

[72] Laparre J, Kaabia Z, Mooney M, Buckley T, Sherry M, Le Bizec B (2017). Impact of storage conditions on the urinary metabolomics fingerprint. Anal Chim Acta.

[73] Teunissen CE, Petzold A, Bennett JL, Berven FS, Brundin L, Comabella M (2009). A consensus protocol for the standardization of cerebrospinal fluid collection and biobanking. Neurology..

[74] Haijes HA, Willemse EAJ, Gerrits J, van der Flier WM, Teunissen CE, Verhoeven-Duif NM, et al. Assessing the pre-analytical stability of small-molecule metabolites in cerebrospinal fluid using direct-infusion metabolomics. Metabolites. 2019;9(10):236. 10.3390/metabo9100236 PMC6835587 31635433

[75] Rosenling T, Slim CL, Christin C, Coulier L, Shi S, Stoop MP (2009). The effect of preanalytical factors on stability of the proteome and selected metabolites in cerebrospinal fluid (CSF). J Proteome Res.

[76] Noga MJ, Zielman R, van Dongen RM, Bos S, Harms A, Terwindt GM (2018). Strategies to assess and optimize stability of endogenous amines during cerebrospinal fluid sampling. Metabolomics..

[77] Saito K, Hattori K, Andou T, Satomi Y, Gotou M, Kobayashi H, et al. Characterization of postprandial effects on CSF metabolomics: a pilot study with parallel comparison to plasma. Metabolites. 2020;10(5):185. 10.3390/metabo10050185 PMC7281358 32384774

[78] Trabado S, Al-Salameh A, Croixmarie V, Masson P, Corruble E, Fève B (2017). The human plasma-metabolome: reference values in 800 French healthy volunteers; impact of cholesterol, gender and age. PLoS One.

[79] Darst BF, Koscik RL, Hogan KJ, Johnson SC, Engelman CD (2019). Longitudinal plasma metabolomics of aging and sex. Aging..

[80] Beyene HB, Olshansky G, AA TS, Giles C, Huynh K, Cinel M, et al. (2020). High-coverage plasma lipidomics reveals novel sex-specific lipidomic fingerprints of age and BMI: evidence from two large population cohort studies. PLoS Biol.

[81] Wu ZE, Fraser K, Kruger MC, Sequeira IR, Yip W, Lu LW (2020). Metabolomic signatures for visceral adiposity and dysglycaemia in Asian Chinese and Caucasian European adults: the cross-sectional TOFI_Asia study. Nutr Metab.

[82] Holmes E, Loo RL, Stamler J, Bictash M, Yap IK, Chan Q (2008). Human metabolic phenotype diversity and its association with diet and blood pressure. Nature..

[83] Minami Y, Kasukawa T, Kakazu Y, Iigo M, Sugimoto M, Ikeda S (2009). Measurement of internal body time by blood metabolomics. Proc Natl Acad Sci U S A.

[84] Kasukawa T, Sugimoto M, Hida A, Minami Y, Mori M, Honma S (2012). Human blood metabolite timetable indicates internal body time. Proc Natl Acad Sci U S A.

[85] Dallmann R, Viola AU, Tarokh L, Cajochen C, Brown SA (2012). The human circadian metabolome. Proc Natl Acad Sci U S A.

[86] Chua EC, Shui G, Lee IT, Lau P, Tan LC, Yeo SC (2013). Extensive diversity in circadian regulation of plasma lipids and evidence for different circadian metabolic phenotypes in humans. Proc Natl Acad Sci U S A.

[87] Karimpour M, Surowiec I, Wu J, Gouveia-Figueira S, Pinto R, Trygg J (2016). Postprandial metabolomics: a pilot mass spectrometry and NMR study of the human plasma metabolome in response to a challenge meal. Anal Chim Acta.

[88] Cao J, Schwichtenberg KA, Hanson NQ, Tsai MY (2006). Incorporation and clearance of omega-3 fatty acids in erythrocyte membranes and plasma phospholipids. Clin Chem.

[89] Nieman DC, Ramamoorthy S, Kay CD, Goodman CL, Capps CR, Shue ZL (2017). Influence of ingesting a flavonoid-rich supplement on the metabolome and concentration of urine phenolics in overweight/obese women. J Proteome Res.

[90] Rothwell JA, Madrid-Gambin F, Garcia-Aloy M, Andres-Lacueva C, Logue C, Gallagher AM (2018). Biomarkers of intake for coffee, tea, and sweetened beverages. Genes Nutr.

[91] Van Dorsten FA, Daykin CA, Mulder TP, Van Duynhoven JP (2006). Metabonomics approach to determine metabolic differences between green tea and black tea consumption. J Agric Food Chem.

[92] Spencer JP (2003). Metabolism of tea flavonoids in the gastrointestinal tract. J Nutr.

[93] Bar N, Korem T, Weissbrod O, Zeevi D, Rothschild D, Leviatan S (2020). A reference map of potential determinants for the human serum metabolome. Nature..

[94] Eriksen R, Perez IG, Posma JM, Haid M, Sharma S, Prehn C (2020). Dietary metabolite profiling brings new insight into the relationship between nutrition and metabolic risk: an IMI DIRECT study. EBioMedicine..

[95] Pietzner M, Kaul A, Henning AK, Kastenmuller G, Artati A, Lerch MM (2017). Comprehensive metabolic profiling of chronic low-grade inflammation among generally healthy individuals. BMC Med.

